# NADPH Oxidases, Angiogenesis, and Peripheral Artery Disease

**DOI:** 10.3390/antiox6030056

**Published:** 2017-07-12

**Authors:** Pradeep Manuneedhi Cholan, Siân P. Cartland, Mary M. Kavurma

**Affiliations:** 1 Heart Research Institute, Sydney 2042, Australia; pradeep.cholan@hri.org.au (P.M.C.); sian.cartland@hri.org.au (S.P.C.); 2 Sydney Medical School, the University of Sydney, Sydney 2006, Australia

**Keywords:** angiogenesis, peripheral artery disease (PAD), NADPH oxidases (NOX), endothelial cell (EC)

## Abstract

Peripheral artery disease (PAD) is caused by narrowing of arteries in the limbs, normally occurring in the lower extremities, with severe cases resulting in amputation of the foot or leg. A potential approach for treatment is to stimulate the formation of new blood vessels to restore blood flow to limb tissues. This is a process called angiogenesis and involves the proliferation, migration, and differentiation of endothelial cells. Angiogenesis can be stimulated by reactive oxygen species (ROS), with NADPH oxidases (NOX) being a major source of ROS in endothelial cells. This review summarizes the recent evidence implicating NOX isoforms in their ability to regulate angiogenesis in vascular endothelial cells in vitro, and in PAD in vivo. Increasing our understanding of the involvement of the NOX isoforms in promoting therapeutic angiogenesis may lead to new treatment options to slow or reverse PAD.

## 1. Introduction

Peripheral artery disease (PAD) is a condition where narrowed arteries reduce blood flow to peripheral arteries, most commonly in the lower extremities. This is a major risk factor for lower-limb amputations [1] and a major risk factor for heart attack [2,3]. Current interventions are insufficient in many PAD patients because extensive disease precludes effective revascularization. This has prompted alternate treatment strategies including angiogenesis, or the stimulation of new blood vessel growth, to restore blood flow and preserve tissue survival.

Angiogenesis is the growth of capillary networks consisting of endothelial cell (EC) tubes, driven by hypoxia-induced mediators; the most characterized being vascular endothelial growth factor (VEGF) [4]. Remodeling, maturation, and stabilization of existing vessels by perivascular cells (pericytes in capillaries) or vascular smooth muscle cells (VSMCs; in larger vessels) are essential for generating functional collateral vessel networks in ischemia. Excessive reactive oxygen species (ROS) can promote oxidative stress, exacerbate atherosclerosis and cardiovascular disease (CVD), however, physiological levels are essential for cell signaling and maintaining homeostasis by their ability to regulate proliferation, migration, and differentiation of cells. Indeed, ROS have been implicated in angiogenesis and stability of the newly formed vessels [5,6,7,8,9,10], and NADPH oxidases (NOXs) are a major source of ROS in ECs. In this review, we summarize the recent progress in understanding ROS-derived NOX, and the role NOXs play in angiogenesis in vitro, and in PAD in vivo.

## 2. NOX Structure and Function

The nicotinamide adenine dinucleotide phosphate (NADPH) system is responsible for generating free radicals in immune and other cells in the body. In humans, 7 isoforms exist, including NOX-1 to -5 and two dual oxidases, DUOX-1 and DUOX-2. Except for NOX-5 and DUOX-1 and -2, NOX are phagocytic oxidases, whose main task is to generate ROS to kill foreign pathogens at homeostasis. They are found in various parts of the body, and each implicated in multiple functions including defense mechanisms, signal transduction, and hormone biosynthesis [11]. Of note, the major source of EC ROS comes from NOX-1, -2, -4 and -5 [12,13]; these isoforms will be the focus of this review.

**Figure 1:**
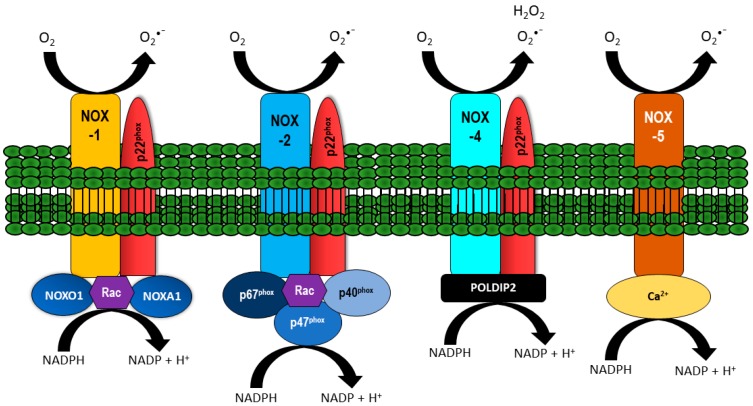
Vascular NADPH oxidase (NOX) and their regulatory subunits. NOX-1, -2, and -4 are localized at the membrane together with p22^phox^. The activity of NOX-1 is regulated by NOXO1, NOXA1, and Rac. NOX-2 activity is dependent on binding p67^phox^, p40^phox^, p47^phox^, and Rac, whilst NOX-4 is constitutively active and can be regulated by polymerase-δ-interacting protein 2 (POLDIP2). NOX-5 activity is influenced by calcium. All NOX isoforms generate O_2_•^−^, with NOX-4 preferentially producing H_2_O_2_.

NOX are transmembrane proteins that transport electrons across the cell membrane to reduce oxygen and produce superoxide (O_2_•^–^). While each NOX contains six transmembrane domains along with a cytoplasmic domain that binds NADPH and flavin adenine dinucleotide, each isoform is differentiated by specific cytosolic and membrane-bound subunits with the prototypic and most characterized being NOX-2 (also known as gp91^phox^). For the prototypic NOX isoform, O_2_•^–^ generation is induced upon assembly of membrane-bound subunits e.g., NOX-2 and p22^phox^, together with cytosolic subunits p67^phox^, p47^phox^, p40^phox^, and a small G-protein, namely Rac. Like NOX-2, NOX-1, and -4 also assemble with p22^phox^, however NOX-1 oxidase activity requires NOX organizer 1 (NOXO1) and NOX activator 1 (NOXA1); isoforms of p67^phox^ and p47^phox^. NOX-4 differs from the prototypic NOX as it is constitutively active, but can be activated by polymerase-δ-interacting protein 2 (POLDIP2). On the other hand, NOX-5 is calcium-dependent and can be regulated by calmodulin [14,15]. NOX-1, -2, -3, and -5 produce O_2_•^–^. NOX-4 also produces O_2_•^–^, but primarily emits H_2_O_2_ [16,17], potentially due to an extended extracytosolic loop that only occurs in the NOX-4 isoform [18]. Structures of NOX-1, -2, -4, and -5 are depicted in Figure 1.

## 3. NOX: Role in Angiogenesis

**Figure 2:**
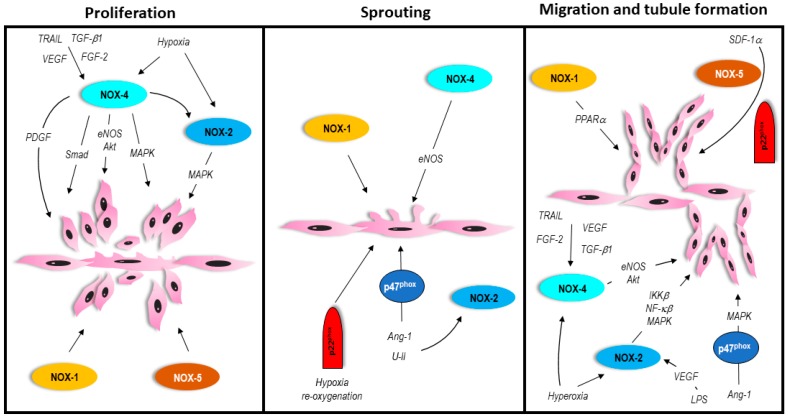
In vitro process of angiogenesis involving vascular NOX isoforms. Please refer to text for details.

Angiogenesis is a normal, important, and carefully controlled process where capillaries form from pre-existing blood vessels. It is driven by pro- and anti-angiogenic factors acting on ECs, and proceeds through several defined stages. Initially, quiescent ECs are activated, stimulating expression of growth factor receptors, and the release of proteases that breakdown the basement membrane. ECs then proliferate and begin sprouting, followed by migration in the direction of the angiogenic stimulus, and form tubules (Figure 2). These events ultimately result in the formation of a lumen. 

eNOS-derived nitric oxide (NO) activity produced by eNOS (which catalyzes the conversion of l-arginine into NO and l-citrulline), is essential in EC homeostasis. Any imbalance or impairment in eNOS-catalyzed NO production/bioactivity leads to a dysfunctional endothelium. Importantly, both eNOS and NO are important mediators of angiogenesis with NO being the effector molecule [19,20]. Though the exact mechanism(s) are not fully understood, NO increases EC proliferation and migration by increasing growth factor levels and expression; growth factors including VEGF, fibroblast growth factor-2 (FGF-2), TNF-related apoptosis-inducing ligand (TRAIL), and urokinase type plasminogen activator (uPA) [19,21,22,23]. Importantly, EC-derived NOX is activated by VEGF, FGF-2, platelet derived growth factor (PDGF), Angiopoietin 1 or -2 (Ang1, Ang2), and TRAIL. NOX are also activated by conditions which induce angiogenesis, namely hypoxia and ischemia, and involve multiple cytokines and enzymes. Here, we will describe the recent advances in NOX isoforms in regulating angiogenic processes in vitro, but also in PAD in vivo. 

### 3.1. In Vitro Process of Angiogenesis

While excessive or supraphysiological levels of ROS can damage and kill ECs [24,25,26,27,28], ROS at physiological levels are essential in mediating regular cellular functions including proliferation, migration, and differentiation [29,30,31]; in vitro processes of angiogenesis. Importantly, these cellular functions have been shown to require NOX isoforms and are described in detail below. 

### 3.2. Proliferation

NOX can produce ROS to inhibit EC proliferation and promote cell senescence in vitro [32,33], however, NOX can also stimulate proliferation and cell survival. The first evidence demonstrating NOX-1’s involvement in cell growth comes from Suh et al. (1999). In this study, NOX-1 overexpression stimulated proliferation of multiple cells, including VSMCs [34], but it wasn’t until recently, that EC proliferation was shown to be regulated by NOX-1, where silencing NOX-1 impaired hypoxia-induced human pulmonary artery EC proliferation [35]. NOX-5 overexpression also promoted human microvascular EC cell growth [36]. While these studies suggest a positive role for NOX-1 and -5 in EC proliferation, mechanism(s) for this are currently lacking and require further elucidation. The majority of the evidence linking NOX’s to EC proliferation comes from work involving NOX-2 and NOX-4. For example, NOX-2 and NOX-4 over expression increased human hybridoma EC proliferation and ROS production [37], but silencing them inhibited ROS and EC proliferation [37]. NOX-4 binding proteins have also been implicated in EC growth; compared to control, siRNA to POLDIP2 inhibited serum-inducible human umbilical vein EC proliferation, suggesting that POLDIP2 directly influences cell cycle processes [38]. Induction of NOX-4 either by NOX-4 overexpression, VEGF [39], FGF-2, TRAIL [40], or transforming-growth-factor-β1 (TGFβ1) exposure [41,42] can stimulate human microvascular EC growth. Importantly, NOX-2 or NOX-4-induced human microvascular EC proliferation appears to involve mitogen activated protein kinase (MAPK), Akt, or Smad pathways [40,43,44]. These pathways can facilitate eNOS activation, nitric oxide or H_2_O_2_ production to stimulate proliferation [40,42,45,46,47]. NOX-4 is also known to interact with phosphorylated VEGF receptor 2 and stimulate VEGF-induced human retinal microvascular EC proliferation [39]. Interestingly, NOX-4 derived H_2_O_2_ can stimulate NOX-2, resulting in VEGF receptor activation, and increased human and mouse EC proliferation [33,42,48]. These findings imply cross-talk between NOX isoforms. Interestingly, both NOX-2 and NOX-4 can increase PDGF receptor protein expression, implying that NOX isoforms may stimulate other growth factors to promote EC proliferation in vitro [49]. Collectively, these studies suggest that NOX-2 and -4 are major contributors to EC growth.

### 3.3. Sprouting

The majority of studies linking NOX isoforms to EC sprouting come from tumor angiogenesis [50,51,52,53,54]. In this review, we have focused on NOX isoforms that mediate vascular EC sprouting, which predominantly include NOX-2 and NOX-4. A study by Craig et al. (2011) [45] demonstrated that aortas from NOX-4 transgenic (NOX-4 TG) mice cultured in Matrigel for 7 days ex vivo, demonstrated ~25% enhanced sprouting compared to wildtype aortas [45]. Importantly, this effect was eNOS dependent, since *eNOS*^−/−^ NOX-4 TG mice did not display enhanced sprouting ability compared to *eNOS*^−/−^ mice alone [45]. Two hours of hypoxia followed by 4 h re-oxygenation also resulted in growth of sprouts in a model of pig coronary artery endothelial spheroids [55]. These effects however, were diminished when p47^phox^ knockout spheroids were exposed to hypoxia/re-oxygenation, implicating NOX cytosolic proteins in sprouting [55]. In a second model, aortas of *p47^phox−/−^* mice had impaired sprouting ability compared to wildtype in response to hypoxia and re-oxygenation [55]. p47^phox^ has also been implicated in Ang-1 [56] and urotensin II (U-II) [57] stimulated sprouting. Ang-1 is an important regulator of vascular angiogenesis [58]. Importantly, in response to Ang-1, pig coronary artery endothelial spheroid sprouting was inhibited by broad-spectrum NOX inhibitors (apocynin or diphenylene iodonium), with *p47^phox−/−^* EC spheroids and aortic ring vessel outgrowths showing modest sprouting compared to wildtype [56]. U-II is a peptide ligand and a potent vasoconstrictor [59]. U-II-induced sprouting in cultured mouse vena cava explants was inhibited by treatment with urantide, a U-II antagonist [57]. Importantly, vascular sprouting of the explants in *Nox2^−/−^* mice was considerably reduced [57]. Furthermore, *Nox2^−/−^* mice had reduced U-II-induced invasion of new vessels into Matrigel plugs [57]. Collectively, these studies suggest that NOX-2 and p47^phox^ are important in EC sprouting processes. 

### 3.4. Migration and Tubule Formation 

The role of NOX-1 in regulating migration and tubule formation has been described [53]. In NOX-1 deficient mouse lung ECs, VEGF, and FGF-2-stimulated intracellular ROS was severely compromised compared to wildtype ECs. Importantly, NOX-1 deficient ECs were unable to migrate in Matrigel, nor could they form tubules on fibrin gel [53]. Interestingly, these EC displayed increased peroxisome proliferator-activated receptor-α (PPARα) expression, and exposure of these cells to GW6471 (PPARα antagonist), restored their ability to migrate and form tubules [53]. While this is suggestive that PPARα may play an antagonistic role in NOX-1-induced angiogenesis, the exact mechanism(s) for NOX-1 mediated tubule formation requires further study. 

Migration of ECs is often initiated upon detection of a hypoxic environment [4,60], but can also be initiated in an environment of excess oxygen. Pendyala et al. (2009) found that hyperoxia-stimulated production of ROS and EC migration, was in part, due to NOX-2 in human lung microvascular endothelial cells [61]. Silencing NOX-2 by adenovirus also inhibited Ang-1-inducible ROS production and tubule formation of human umbilical vein EC’s [62]. Moreover, Ang-1-induced migration was impaired in ECs isolated from *p47^phox−/−^* mice, associating with reduced intracellular ROS and reduced activation of Akt and p42/44 MAP kinase [55]. The role of Ang-2 in angiogenesis has also been described. Here, lipopolysaccharide (LPS)-induced VEGF and Ang-2 expression resulted in increased human pulmonary microvascular EC ROS production, however, only inhibition of NOX-2 by siRNA, but not NOX-1 and -4, reduced O_2_•^–^ levels [63]. LPS also stimulated the formation of EC tubules, which was attenuated by NOX-2, involving IκB kinase-β (IKKβ)/NF-κB and MAPK/AP-1 pathways [63].

Like NOX-2, NOX-4 can promote EC migration and tubule formation. A role for NOX-4 in hyperoxia has been identified [61]. Here, the authors found that hyperoxia-induced migration and tubule formation of human lung ECs in vitro, was significantly attenuated when NOX-2 and NOX-4 were silenced [61]. In addition to hyperoxia, the role of NOX-4 has been further examined in response to other factors. For example, H_2_O_2_ production and TGFβ1-induced capillary formation was abolished when NOX-4 was silenced in ECs [41]. Silencing NOX-4 also inhibited intracellular ROS production, attenuated eNOS phosphorylation, as well as impaired TRAIL-inducible human microvascular EC migration and tubule formation [40]. Stromal cell-derived factor-1 (SDF-1α) is a potent angiogenic chemokine and induces migration of human microvascular EC [64]. When p22^phox^ or NOX-5 were silenced by siRNA, migration induced by SDF-1α was inhibited [65]. Silencing of p22^phox^ and NOX-5 subunits also significantly reduced SDF-1α-induced tubule formation after 72 h. This suggests that multiple NOXs with p22^phox^ subunits and NOX-5, are involved in SDF-1α migration [65].

### 3.5. In Vivo Processes of Angiogenesis in PAD

Hindlimb ischemia is a common model of PAD. In most cases, this model involves ligation and excision of the femoral artery and all side branches, with the ischemic process naturally promoting angiogenesis in normal mice [66]. However, variations to this model do exist, and can affect angiogenic outcomes [67]. For example, cutting or partial/complete resection of the femoral artery, iliac artery, or femoral vein, independently, or collectively, or injury via coagulation, can alter the degree of ischaemic damage, affecting degree of vascularity and limb perfusion [67,68]. As described above, NOX isoforms are upregulated in response to hypoxic conditions and implicated in angiogenic processes in vitro. These suggest that NOX may play a role in angiogenesis in response to ischemic injury in vivo. However, to date only NOX-2 and NOX-4 have been proven to control the angiogenic process in PAD following hindlimb ischemia. For example, NOX-2 protein expression was significantly increased in newly formed capillaries and leukocytes in the ischemic limbs of wildtype mice [69]. Hindlimb ischemia also increased NOX-2, -4, and p47^phox^ mRNA expression in collateral arteries of rats at day 1, 3, and 7 post-surgery, whereas NOX-1 expression was undetected [70]. The importance of NOX-2 in stimulating angiogenesis in PAD was confirmed in *Nox2^−/−^* mice; blood perfusion was significantly impaired 7 days after ischemic injury when compared to wildtype, associating with reduced capillary density and reduced O_2_•^–^ production, but not inflammation [69]. Reduced collateral vessel growth was also observed in rats where apocynin treatment or NOX-2 inhibition suppressed angiogenesis, and inhibition of p47^phox^ by siRNA in rats, or in *p47^phox−/−^* mice, led to reduced collateral vessel growth in response to ischemia [70]. The bone marrow has also been implicated in NOX-2-induced angiogenesis in PAD. ROS production, hypoxia, and HIF-1α expression were increased in bone marrow after ischemic injury; a finding not observed in *Nox2^−/−^* mice [71]. Importantly, reduced ROS in bone marrow of *Nox2^−/−^* mice associated with impaired vascularity and blood flow, as well as reduced endothelial progenitor cell numbers in peripheral blood [71]. The authors concluded that NOX-2 dependent hypoxia in bone marrow stimulates EC progenitor expansion to facilitate mobilization of these cells to areas of ischemic injury for tissue repair [71]. In support, more recent studies suggest that EC-specific lineage and angiogenic potency of progenitor stem cells in mice require NOX-2 [72]. While these studies imply that NOX-2 is crucial in stimulating angiogenesis in PAD, other studies contrastingly demonstrate no change in blood perfusion or vascularity in *Nox2^−/−^* mice [45,73]. It is unclear as to why there are discrepancies, however, variations in the way ischemia was initiated is different between studies, confirming that variations to the model can result in differences in vascularization [67,68]. 

Like NOX-2, NOX-4 can promote angiogenesis after hindlimb ischemia. This has been demonstrated using multiple models in vivo including NOX-4 adenoviral gene therapy, and using transgenic and *Nox4^−/−^* mice [45,74]. For example, local adenoviral NOX-4 injection 3 days prior to ischemia improved blood flow recovery and stimulated capillary density in mice 28 days after injury [45]. A similar finding was observed in EC-specific NOX-4 transgenic mice [45]. Not only did vascular tissue from these mice display enhanced capillary sprouting ex vivo, in response to hindlimb ischemia, these mice had enhanced capillary density in skeletal muscle, and marked improvement in blood perfusion compared to littermate control mice [45]. Importantly the authors showed that eNOS activation was critical for NOX-4-induced angiogenesis in PAD [45]. In contrast, global *Nox4^−/−^* or tamoxifen-inducible deletion of NOX-4 in mice significantly attenuated blood flow recovery and vascularity in response to hindlimb ischemia [74]. Furthermore, a reduction in NOX-4 mRNA expression in skeletal muscle 3 days after hindlimb ischemia was observed in *Trail^−/−^* mice associating with impaired blood flow and capillary density [40]. Importantly, NOX-4 deletion in mice also resulted in reduced eNOS and nitric oxide production [74]. *Poldip2^−/−^* mice are embryonic lethal [75], however, *Poldip2^+/−^* mice exhibited reduced blood perfusion to the lower hindlimb, associating with reduced capillary density, macrophage infiltration, and ~44% reduction in H_2_O_2_ production in gastrocnemius muscle after ischaemic injury [38]. The authors proposed that new collaterals and maintenance of new vascular networks were dependent on POLDIP2, since *Poldip2^+/−^* mice displayed regressed vasculature [38]. Using the porcine small intestine submucosa implant model of angiogenesis, *Poldip2^+/−^* mice also had reduced endothelial invasion by approximately 50%. Collectively, these demonstrate the importance NOX-2 and -4 and their subunits in angiogenic responses in vivo.

### 3.6. NOX-Mediated Perivascular Cell Processes Contributing to Vessel Stability

Perivascular cells including pericytes and VSMCs, play important roles in the stability of new blood vessels. Stabilization and maturation of the newly-formed vessels is achieved by the activation, proliferation, and recruitment of these cells, which wrap around the EC-tubes, providing support and maintenance [76,77,78]. Pericytes closely associate and are in direct contact with small vessels such as capillaries or microvasculature, whereas VSMCs wrap around larger arteries and veins, however, they are separated by an extracellular matrix and are not in direct contact with the endothelium [79]. 

NOX-4 was identified as the main source of ROS in human brain pericytes, since NOX-4 RNAi, but not rotenone, l-Name, or oxypurinol inhibited ROS production [80]. Importantly, silencing NOX-4 also blocked basal proliferation of these cells [80]. While this study implicates NOX-4-mediated ROS on pericyte proliferation, the majority of work involving NOX isoforms in regulating proliferation and migration in perivascular cells comes from VSMCs. For example, NOX-1 expression was increased within 3 d after carotid artery ligation in Sprague Dawley rats, associating with elevated O_2_•^–^ in vessels derived from intimal and medial VSMCs [81]. The role of NOX-1-induced ROS in VSMC proliferation and migration in vivo was further confirmed using mice with global NOX-1 deletion (*Nox1^−/y^*), or mice expressing NOX-1 specifically in VSMCs (*Tg^SMCnox1^*) [82]. *Nox1^−/y^*, but not *Tg^SMCnox1^* mice had reduced intimal thickening in response to femoral artery wire injury [82]. The role of NOX-1 was further characterized in VSMCs isolated from aortas of wildtype, *Nox1^−/y^* and *Tg^SMCnox1^* mice. Importantly, *Tg^SMCnox1^* VSMCs exhibited increased PDGF-induced O_2_•^–^ production, associating with increased growth rate and migration [82]. Multiple factors have been shown to regulate VSMC processes via NOX. These include epidermal growth factor receptor [83], FGF-2 [84], AngII [85], and the plasminogen/plasmin system; important in blood coagulation and known to participate in vascular remodeling [86]. Of note, uPA-inducible ROS ranged from 20 to 80 nmol/L in VSMCs; and using pharmacological or molecular approaches, inhibition of NOX-1 significantly reduced ROS generation and aortic VSMC proliferation [86]. Reports suggest NOX-2 is expressed in VSMCs [87,88], however, its role in processes relating to angiogenesis is not fully defined, whereas, NOX-4 has been implicated. For example, silencing NOX-4 reduced human VSMC proliferation and increased senescence [89], implicating a role for NOX-4 in VSMC growth. NOX-4 also stimulated VSMC differentiation [90], and affected VSMC integrity and migration, in part via POLDIP2 [91]. Here, silencing POLDIP2 reduced intracellular O_2_•^–^ and H_2_O_2_, as well as POLDIP2, NOX-4, and p22^phox^ protein expression, with marked reduction in the localization of these proteins to focal adhesions in rat thoracic aortic VSMCs [92]. Of note, silencing NOX-4 had a similar effect on cytoskeletal integrity [92]. Consistent with these, *Poldip2^+/−^* mice had reduced VSMC content in ischemic skeletal muscle, suggesting impaired VSMC remodeling in response to hindlimb ischemia [38]. Finally, reports linking NOX-5 to VSMC proliferation have been described [93]. SiRNA to NOX-5 significantly reduced PDGF-induced O_2_•^–^ production, from ~800 to 500 pmol/mg protein (as assessed by 2-hydroethidium and HPLC) and VSMC proliferation via the janus kinase (JAK)/signal transducer and activator of transcription (STAT) pathway [93]; however, the precise mechanisms for this are unknown. Collectively, these studies provide insight into NOX signaling in perivascular cell processes related to angiogenesis. More studies are required to understand the role of NOX in differentiation, proliferation, and migration of perivascular cells, and how they may contribute to vessel stability in angiogenesis.

## 4. NOX—Lessons from the Clinic

PAD is often a consequence of atherosclerosis, and NOX-derived oxidative stress has been linked to lesion progression [94,95,96,97]. Of note, pharmacological inhibition of NOX can block ROS production, thereby reducing atherosclerotic activity and disease progression in preclinical models [98,99]. These suggest that drugs targeting NOX may be beneficial in atherosclerosis and in people with PAD. Importantly, NOX subunit p22^phox^ expression was increased in atherosclerotic vs. non-atherosclerotic arteries [100]. NOX-2 and NOX-4, but not NOX-1 were found to be abundant in diseased arteries, correlating with atherosclerosis severity [101]. The first evidence of NOX as a major source of O_2_•^–^ in atherosclerotic blood vessels was described by Guzic et al. (2000) [102]. This has since been confirmed by others, where O_2_•^–^ was localized to coronary arteries and increased in atherosclerotic plaque shoulders, associating with NOX-2, p22^phox^, and to some extent, NOX-4 expression [101]. Notably, NOX-2 and p22^phox^ mRNA was increased with the severity of disease [101]. Differential expression of NOX isoforms dependent on the vessel bed have also been described, with veins expressing more NOX-2 and p22^phox^, and arteries expressing more NOX-4 [103] Importantly, a strong correlation between O_2_•^–^ production with p22^phox^ and NOX-4; NOX activity and endothelial dysfunction was observed [103]. Very little has been described in relation to PAD, with one study showing increased plasma NOX-2 associating with impaired endothelial function [104]. Interestingly, dark chocolate, reduced markers of oxidative stress including circulating NOX-2, and improved walking autonomy in patients with PAD diagnosed with intermittent claudication [105]. While these studies suggest that NOX isoforms may be important mediators of ROS in artery dysfunction and cardiovascular diseases, evidence that NOX-mediated ROS increases atherosclerotic risk in people is still lacking or controversial e.g., in chronic granulomatous disease, an X-linked immune-deficiency disease caused by mutation in one or all NOX-2 components. While, these patients have profound bacterial and fungal infection due to the inability to eliminate foreign pathogens [106,107], they have reduced carotid atherosclerosis [108], raising questions of the in vivo role of NOX-2 in CVD. The role of NOX isoforms in angiogenesis in vascular diseases are also unclear. As such, more studies are needed to fully elucidate the role of NOX-mediated ROS in vascular disease. 

## 5. Conclusions

PAD can lead to gangrene and potential limb amputation. While current treatments include lifestyle changes and stent placement, a new approach is to increase vascularization in the affected limb by targeting ECs and stimulating angiogenesis. At physiological levels EC-derived ROS is pro-angiogenic. In vitro studies have implicated NOX in important stages of angiogenesis; proliferation, sprouting, migration, and tubule formation. Furthermore, pre-clinical in vivo studies have confirmed the importance of NOX family members in reperfusion after hind limb ischemia. Crucially, clinical investigations have shown changes in NOX expression in the vessels of patients diagnosed with PAD, suggesting these isoforms may be beneficial as therapeutic targets. Increasing our understanding of the mechanism(s) regulating NOX-induced angiogenesis will be highly beneficial in the development of treatments to increase both lifespan and life quality for patients with PAD.
